# Impact of diabetes mellitus on life expectancy and health-adjusted life expectancy in Canada

**DOI:** 10.1186/1478-7954-10-7

**Published:** 2012-04-24

**Authors:** Lidia Loukine, Chris Waters, Bernard CK Choi, Joellyn Ellison

**Affiliations:** 1 Centre for Chronic Disease Prevention and Control, Public Health Agency of Canada (PHAC), Government of Canada, Ottawa, ON, Canada; 2 Department of Epidemiology and Community Medicine, University of Ottawa, Ottawa, ON, Canada; 3 Shantou University Medical College, Shantou, China; 4 Centre for Chronic Disease Prevention and Control, Public Health Agency of Canada, Government of Canada, A.L.6806A, 785 Carling Avenue, Ottawa, ON, K1A 0 K9, Canada

**Keywords:** Life expectancy, Health-adjusted life expectancy, Diabetes mellitus, Health utilities index, Summary measure of population health

## Abstract

The objectives of this study were to estimate life expectancy (LE) and health-adjusted life expectancy (HALE) for Canadians with and without diabetes and to evaluate the impact of diabetes on population health using administrative and survey data.

Mortality data from the Canadian Chronic Disease Surveillance System (2004 to 2006) and Health Utilities Index data from the Canadian Community Health Survey (2000 to 2005) were used. Life table analysis was applied to calculate LE, HALE, and their confidence intervals using the Chiang and the adapted Sullivan methods.

LE and HALE were significantly lower among people with diabetes than for people without the disease. LE and HALE for females without diabetes were 85.0 and 73.3 years, respectively (males: 80.2 and 70.9 years). Diabetes was associated with a loss of LE and HALE of 6.0 years and 5.8 years, respectively, for females, and 5.0 years and 5.3 years, respectively, for males, living with diabetes at 55 years of age. The overall gains in LE and HALE after the hypothetical elimination of prevalent diagnosed diabetes cases in the population were 1.4 years and 1.2 years, respectively, for females, and 1.3 years for both LE and HALE for males.

The results of the study confirm that diabetes is an important disease burden in Canada impacting the female and male populations differently. The methods can be used to calculate LE and HALE for other chronic conditions, providing useful information for public health researchers and policymakers.

## Background

In 2006, approximately 2 million Canadians aged 1 year and older (6.2% of the total population) had diagnosed diabetes (type 1 and type 2) [1]. The number of Canadians with diagnosed diabetes increased by about 651,000 for the period of 2001 to 2006 and is projected to reach almost 2.8 million in 2012. Diabetes increases the risk of developing other life-threatening diseases such as heart attack, stroke, or kidney failure. This leads to poor health, premature mortality, and to a reduction of life expectancy (LE) and health-adjusted life expectancy (HALE). Even though the mortality among people with diagnosed diabetes is decreasing due to better diabetes care, it still remains high. The decrease in mortality means an increase in longevity but does not necessarily lead to an increase of the number of healthy years in a person’s life. Over the long term, living with diabetes decreases quality of life and increases the use of health care services. Therefore, it is important to monitor the gap between LE and HALE to see if programs and policies are positively impacting a life in good health by narrowing the gap or if modifications are required.

HALE is a summary measure of population health (SMPH). While LE is the average number of years a person is expected to live, HALE is life expectancy weighted or adjusted for the level of health-related quality of life (HRQOL). Morbidity and mortality data are combined into one single indicator of population health that indicates the average time that a person could expect to live in full health. A comparison of disparities in LE and HALE for populations of people with and without diabetes, an assessment of the loss and gain in LE and HALE, and the proportion of life lived in poor health among different cohorts can provide a comprehensive picture of the current impact of diabetes.

Very few studies provide estimates of the impact of diabetes on population health using a SMPH. In Canada, Manuel et al. [2] and Sikdar et al. [3] estimated diabetes-deleted life expectancy and the gain in LE after a hypothetical removal of diabetes-related deaths using a cause-deleted method. Results for LE and HALE for individuals with and without diabetes were published for Ontario, a province of Canada, by Manuel et al. [4-6]. Different aspects of diabetes-related life expectancy were studied in the United States. Narayan et al. estimated the lifetime probability of developing diabetes, life-years lost, and quality-adjusted life years (QALYs) lost associated with diabetes using a Markov chain model [7]. Cunningham et al. estimated diabetes–free life expectancy using the multiple-decrement life table method [8].

This is the first population-based study of the impact of diabetes on LE and HALE in Canada using administrative data collected by the Canadian Chronic Disease Surveillance System (CCDSS) and Canadian Community Health Survey (CCHS) data. We estimated LE and HALE for individuals with and without diagnosed diabetes, the gain in LE after hypothetical elimination of diabetes using a proposed disease-deleted method, and the loss in LE and HALE associated with diagnosed diabetes in Canada.

## Data sources and methods

HALE is a composite indicator that combines morbidity and mortality into a single statistic. Mortality data from the CCDSS were used to estimate age- and sex-specific mortality rates. While mortality and population counts are sufficient for calculating LE, a measure of HRQOL is also needed to estimate HALE, its variance, and corresponding 95% confidence intervals. This measure was estimated by using the Health Utilities Index Mark 3 (HUI3) from the CCHS. The HUI3 is a suitable instrument for population-based health status evaluation for type 2 diabetes [9].

### Distinguishing Canadians with and without diagnosed diabetes and estimating their mortality rates

The CCDSS is a collaborative network of provincial and territorial chronic disease surveillance systems, supported by the Public Health Agency of Canada [10]. It was created to broaden the scope of information about the burden of chronic diseases in Canada so that policymakers, researchers, health practitioners, and the general public could make better public and personal health decisions. The CCDSS uses data from various population-based sources in order to estimate the prevalence, incidence, mortality, and the utilization of health care services related to diabetes and other chronic diseases.

In each province and territory, the health insurance registry database is linked to the physician billing and hospitalization databases to identify evidence of diabetes care for residents of Canada who have used the Canadian health care system. The CCDSS represents almost the entire Canadian population, excluding full-time members of the Canadian Forces, the Royal Canadian Mountain Police, and individuals in federal correctional facilities. It is assumed that a person had diagnosed diabetes if there is sufficient evidence of use of the health care system due to diabetes. The minimum requirement is at least one hospitalization or two physician claims over a two-year period with the specific code(s) for diabetes in the International Classification of Diseases (ICD) (ICD-9 codes 250; ICD-10 codes E10–E14) [11,12]. The CCDSS collects data for Canadians aged 1 year and older.

For Canadians with and without diagnosed diabetes, age-specific mortality rates for all causes of death were estimated using the CCDSS. Age-specific mortality rates for persons with and without diagnosed diabetes were used to calculate LE and HALE.

### Measuring HRQOL

As a measure of HRQOL, the HUI3 from three CCHS data files were used for this study: (1) cycle 1.1 2000/2001 share file [13]; (2) cycle 2.1 2003 subsample 1 file [14]; and (3) cycle 3.1 2005 subsample 1 file [15].

The CCHS is a cross-sectional national health survey conducted by Statistics Canada [13-15] that collects information related to the health status, health care utilization, and health determinants for the Canadian population. It includes a sample of about 130,000 respondents and is designed to provide reliable estimates at the local health region level.

Prior to 2007, data collection occurred every two years for a 12-month period. After major changes to the survey design in 2007, data collection now occurs on an ongoing (monthly) basis with annual releases. Data are available for 2000/2001, 2003, 2005, 2007, 2008, and 2009.

The CCHS produces an annual microdata file and a file combining two years of data. The survey years can also be combined to examine subpopulations of rare characteristics. Respondents to the survey were asked if they have any of 26 to 30 chronic conditions, including diabetes. The survey includes respondents aged 12 years and older. The survey does not include people who live in institutions or in remote areas. The household-level response rate in 2005 was 84.9%, and the person-level response rate was 92.5% [15].

HUI3 is a multi-attribute utility measure that defines health states according to eight attributes (vision, hearing, speech, ambulation, dexterity, emotion, cognition, and pain), with five or six levels ranging from normal to severely limited functioning for each. Single attribute utility scores range from 0.0 (lowest level of functioning) to 1.0 (full functional capacity) [16,17]. The eight attributes are combined into a single score using the multi-attribute utility function:

(1) $$u=1.371*\left(b_{1}*b_{2}*b_{3}*b_{4}*b_{5}*b_{6}*b_{7}*b_{8}\right)-0.371$$

where *u *is HUI3 and $b_{i}$ is a single attribute utility score.

The overall scores on the HUI3 range from −0.36 (the worst possible HUI3 health state) through 0.0 (death) to 1.0 (perfect health). From a societal perspective, some health states are considered worse than dead, and consequently are assigned negative scores. Differences of 0.03 or more in overall HUI3 scores and 0.05 or more in single-attribute utility scores are considered to be clinically important [17].

Sex- and age-specific HUI3 averages were estimated from a combined CCHS data file for all respondents and separately for persons with and without diabetes. The bootstrap methodology [18] recommended by Statistics Canada was used to calculate the variance estimate. Both the point estimates and variance estimations were calculated using the BOOTVAR_V31 macro [19]. The HUI3 data for the three youngest age groups (<1, 1–4, and 5–9 years) were not available. A value of 0.999 was used for those age groups on the presumption that not everyone in those age groups had perfect health. To assess the robustness of the assigned HUI3 weight of 0.999, two other HUI3 values, 0.90 and 1.00, were also used in a sensitivity analysis to examine the effect on the HALE calculations.

### Survey sample sizes

All three cycles of the CCHS (cycle 1.1 share file, cycle 2.1 subsample, and cycle 3.1 subsample) were combined by the pooled method to increase the sample size and to decrease variation in the estimates [20]. The sample size for the combined file, which included people 12 years or older and covered the 2000–2005 period, was 200,809 (190,271 without diabetes; 10,538 with diabetes).

Mortality data for Quebec and Nunavut were unavailable from the CCDSS, and Health Utilities Index data from the Northwest Territories and Nunavut were unavailable from the 2000/2001 CCHS (cycle 1.1) survey file. Therefore, these jurisdictions were excluded from the analyses.

### Calculating LE

The Chiang method [21] was used to generate period (2004–2006) life tables by disease-specific/disease-deleted populations and sex using 19 standard age groups (<1, 1–4, 5–9, …, 80–84, 85+ years). The Gompertz function was used to provide an accurate estimate of LE for the last open-ended 85+ age interval in order to close the life table. This method was described by Hsieh [22]. The modified Sullivan method [23] was applied for the HALE calculation. According to this method the “life-years lived” was adjusted by the HUI3.

(2) $$L_{x}^{'}=L_{x}*HUI3_{x}$$

Where $L_{x}^{'}$ is adjusted life-years lived in age-interval x, $L_{x}$ is life-years lived in age-interval x, and HUI3_x_ is Health Utilities Index Mark 3 for people in age-interval x.

The variance of LE was calculated by the Chiang method. The variance of HALE for the population without diabetes was calculated by the Bebbington method [24], accounting only for the variability of HUI3. The sample sizes of the total population and population without diabetes were large, therefore the variance of the life table was close to zero and did not contribute much to the variance of HALE. However, the sample size of the population with diabetes was relatively small and the variance had to be calculated differently. The separate calculation accounted for both the variability of the life table and the variability of HUI3. This method was introduced by Mathers [25]. All calculations were performed using specially developed SAS macros. The 95% confidence intervals for LE and HALE were built based on the normality assumption. As this study has a large sample size, no sensitivity analysis was deemed necessary for the normality assumption. Z-tests were used to test the statistical significance of the loss and gain in life expectancy and the absolute difference in life expectancy.

### Calculating the gain in LE or HALE

The gain in LE (or HALE) after the hypothetical elimination of diagnosed diabetes was calculated by disease-deleted method. According to this method the gain in LE (or HALE) is the difference in LE (or HALE) between the population without diagnosed diabetes and the total population.

## Results

**Figure 1:**
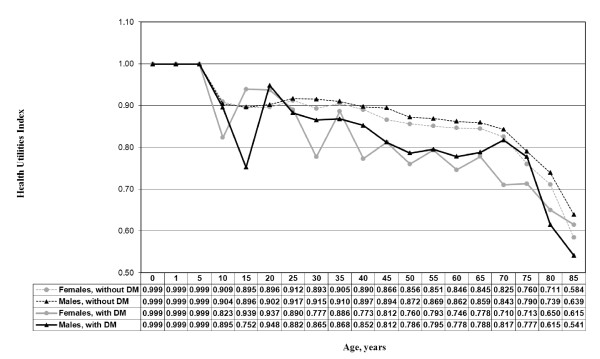
^**1**^**Data source: Canadian Community Health Survey Data Files (CCHS) from Statistics. Canada, 2000–2005.** Dataset for this study excluded Quebec, Nunavut, and Northwest. Territories.

Figure1 illustrates the differences in age-and sex-specific average HUI3 for persons with and without diabetes. The HUI3 scores reported by males were in general higher than those reported by females across all age groups in the population without diabetes, reflecting the differences in morbidity between males and females [26,27]. A similar pattern was observed for individuals with diabetes who were younger than 80 years. For individuals 80 years and older, the pattern reversed and there was a drop in HUI3 for males compared with that for females. The variance of HUI3 estimates was large resulting from a small sample size for the population of people with diabetes, especially for the oldest age groups.

The difference in LE between female and male populations with diagnosed diabetes varied between 4.3 and 1.6 years across age groups, and the difference in HALE was between 2.1 and 1.4 years, lower than that in the population without diabetes (Table 1). Differences in HALE between sexes were approximately half the difference in LE. The narrower difference in HALE was a result of males reporting better health (higher HUI3 score) than females. Many differences in LE and HALE reported in Table 1 were statistically significant.

**Table 1:** **Years difference in life expectancy (LE) and health-adjusted life expectancy (HALE) between females and males with and without diabetes (DM) for selected ages, Canada, 2004 -2006**^**1**^

	**LE(F)-LE(M)**	**HALE(F)-HALE(M)**
**At birth**		
Without DM	4.8*	2.4*
With DM	4.0*	2.1
**At age 20**		
Without DM	4.7*	2.2*
With DM	4.3*	1.7
**At age 55**		
Without DM	4.0*	1.9*
With DM	3.0*	1.4**
**At age 80**		
Without DM	2.3*	1.0*
With DM	1.6**	1.5**

* Statistically significant (p-value <0.0001).

** Statistically significant (p-value <0.05).

^1^ Dataset for this study excluded Quebec, Nunavut, and Northwest Territories.

Table 2 shows age- and sex-specific estimates of the total (LE) and the healthy (HALE) number of years expected to live among individuals without and with diabetes and among the total Canadian population at selected ages. The reasons for the selected ages were the following: comparability with other research on HALE (at birth); examination of the adult population (age 20); the CCDSS mean age at diagnosis of diabetes (age 55); and the last closed age interval in our life tables (age 80). LE and HALE at birth are summary measures and were estimated based on the mortality experience of people with and without diabetes for the period of 2004 to 2006. The definition of "at birth" in this study requires qualifications. First, diabetes does not occur at birth. Second, the CCDSS only collects data for individuals who are 1 year or older. Therefore, the with “diabetes at birth estimates” represent people who had diagnosed diabetes by the age of 1 year and the mortality experience of people with diagnosed diabetes living in that period. The table also includes an estimate of the LE and HALE loss associated with diabetes and the gain realized by hypothetically eliminating diagnosed diabetes from a population.

**Table 2:** **Life expectancy (LE), health-adjusted life expectancy (HALE) (with 95% confidence intervals), and years lost in LE and HALE at selected ages, by diabetes (DM) status and sex, Canada, 2004-2006**^**1**^

**Sex**	**Measure**	**Total population** **(I)**	**Without DM** **(II)**	**With DM** **(III)**	**Loss of LE** **associated with DM** **(II-III)**	**Gain in LE after eliminating DM** **(II-I)**
**At birth**						
**Females**	LE	83.6(83.6-83.7)	85.0(85.0-85.1)	74.9(74.3-75.6)	10.1^*^(9.5-10.7)	1.4^*^
	HALE	72.1(71.8-72.3)	73.3(73.0-73.5)	62.2(60.7-63.8)	11.1^*^(9.5-12.7)	1.2^*^
	LE-HALE^2^		11.7^*^ (11.4-12.0)	12.7^*^(11.8-13.6)		
	(LE-HALE)/LE^3^		0.14	0.17		
**Males**	LE	78.9(78.8-78.9)	80.2(80.2-80.3)	70.9(70.4-71.4)	9.3^*^(8.8-9.8)	1.3^*^
	HALE	69.6(69.4-69.9)	70.9(70.7-71.2)	60.1(58.4-61.9)	10.8^*^(9.1-12.5)	1.3^*^
	LE-HALE^2^		9.3^*^(9.1-9.5)	10.8^*^(9.6-12.0)		
	(LE-HALE)/LE^3^		0.12	0.15		
**At age 20**						
**Females**	LE	64.3(64.2-64.3)	65.7(65.7-65.7)	56.5(56.2-56.8)	9.2^*^(8.9-9.5)	1.4^*^
	HALE	53.6(53.3-53.9)	54.8(54.5-55.1)	44.7(43.4-46.1)	10.1^*^(8.7-11.5)	1.2^*^
	LE-HALE^2^		10.9^*^(10.6-11.2)	11.8^*^(10.8-12.8)		
	(LE-HALE)/LE^3^		0.17	0.21		
**Males**	LE	59.6(59.6-59.7)	61.0(61.0-61.1)	52.2(51.9-52.5)	8.8^*^(8.5-9.1)	1.4^*^
	HALE	51.3(51.1-51.6)	52.6(52.3-52.9)	43.0(41.9-44.1)	9.6^*^(8.5-10.7)	1.3^*^
	LE-HALE^2^		8.4^*^(8.2-8.6)	9.2^*^(8.4-10.0)		
	(LE-HALE)/LE^3^		0.14	0.18		
**At age 55**						
**Females**	LE	30.6(30.6-30.6)	32.0(31.9-32.0)	26.0(25.9-26.1)	6.0^*^(5.9-6.1)	1.4^*^
	HALE	23.6(23.4-23.9)	24.7(24.4-25.0)	18.9(18.3-19.6)	5.8^*^(5.1-6.5)	1.1^*^
	LE-HALE^2^		7.3^*^(7.1-7.5)	7.1^*^(6.5-7.7)		
	(LE-HALE)/LE^3^		0.23	0.27		
**Males**	LE	26.8(26.7-26.8)	28.0(28.0-28.1)	23.0(23.0-23.1)	5.0^*^(4.9-5.1)	1.2^*^
	HALE	21.6(21.4-21.8)	22.8(22.5-23.1)	17.5(17.0-18.1)	5.3^*^(4.7-5.9)	1.2^*^
	LE-HALE^2^		5.2^*^(5.0-5.4)	5.5^*^(5.0-6.0)		
	(LE-HALE)/LE^3^		0.19	0.24		
**At age 80**						
**Females**	LE	11.0(10.9-11.0)	11.7(11.7-11.7)	9.1(9.1-9.2)	2.6^*^(2.6-2.6)	0.7^*^
	HALE	7.0(6.7-7.2)	7.4(7.1-7.7)	5.8(5.2-6.3)	1.6^*^(0.9-2.3)	0.4^*^
	LE-HALE^2^		4.3^*^(4.0-4.6)	3.3^*^(2.8-3.8)		
	(LE-HALE)/LE^3^		0.37	0.36		
**Males**	LE	8.8(8.7-8.8)	9.4(9.3-9.4)	7.5(7.4-7.5)	1.9^*^(1.9-1.9)	0.6^*^
	HALE	5.9(5.6-6.2)	6.4(6.1-6.8)	4.3(3.7-5.0)	2.1^*^(1.4-2.8)	0.5^**^
	LE-HALE^2^		3.0^*^(2.7-3.3)	3.2^*^(2.6-3.8)		
	(LE-HALE)/LE^3^		0.32	0.43		

* Statistically significant (p-value <0.0001).

** Statistically significant (p-value <0.05).

^1^Dataset excluded Quebec, Nunavut, and Northwest Territories.

^2^LE-HALE: years difference between life expectancy and health-adjusted life expectancy.

^3^(LE-HALE)/LE: the proportion of life spent in poor health.

As expected, higher values for both LE and HALE were obtained for people not diagnosed and who did not report diabetes. For females at birth who were not diagnosed with diabetes, LE was estimated to be 85.0 years and HALE was 73.3 years. The corresponding LE and HALE for males was 80.2 and 70.9 years, respectively. LE and HALE for people with diabetes were significantly lower: 74.9 and 62.2 years for females, respectively; and 70.9 and 60.1 years for males, respectively. For 55-year-old females with diabetes, the remaining LE was 26.0 years and HALE was 18.9 years. The corresponding LE and HALE for males were 23.0 and 17.5 years, respectively. Within the population of people without diabetes at this age, the LE and HALE were, respectively, 32.0 and 24.7 years for females and 28.0 and 22.8 years for males. The results at birth reported in this study had a similar pattern as the results obtained by Manuel et al. for Ontario [4,5].

Table 2 also illustrates a number of years and the proportion of life spent in poor health including the loss in LE and HALE associated with diabetes and the gain in LE and HALE after the hypothetical elimination of diabetes within age-sex grouping by disease status.

The number of years spent in poor health is the difference between LE and HALE, and its ratio to the number of total years expected to live can be interpreted as the portion of life spent in poor health. It was observed that, at birth, both measures were greater for persons with diabetes than for persons without diabetes. The number of years individuals with diabetes spent in poor health decreased and the portion of life they spent in unhealthy states increased with increasing age. Females with diabetes who were younger than 55 years spent a greater number of years in poor health than did females of that age without the disease. The opposite pattern was observed for females 55 and older. This phenomenon can be explained by the fact that the HUI3 of those females without diabetes declines faster with age than the HUI3 of females with diabetes.

The loss (see column six in Table 2) of total (LE) or healthy (HALE) life expectancy associated with diabetes was defined in the study as a difference in LE or HALE between populations of people without and with diabetes. Comparing LE and HALE between these two cohorts allows estimating the impact of diabetes on the population and discovering who was affected most. The loss in LE and HALE displays the estimated total and healthy number of years that people with diabetes lose. In this study the loss in LE varied from 10.1 years at birth to 2.6 years at age 80 for females and from 9.3 to 1.9 years for males. The corresponding loss in HALE ranged from 11.1 to 1.6 years for females and from 10.8 to 2.1 years for males. The age-specific life expectancy losses followed a pattern similar to the number of years spent in poor health. That is, the loss in LE was less than the loss in HALE for females younger than 55 years. For the older age groups the loss in LE was greater than the loss in HALE.

The gain in LE and HALE after a hypothetical elimination of diagnosed diabetes from a population is the difference in LE and HALE between the population without diabetes and the total population. The gain in LE at birth was estimated as 1.4 years for females and 1.3 years for males. The corresponding gain in HALE was 1.2 and 1.3 years for females and males, respectively.

The number of years spent in poor health and the losses and gains in LE and HALE were statistically significant for both sexes and all ages (p-value <0.05).

Table 3 shows results of a sensitivity analysis to assess the robustness of the assigned HUI3 value for the three youngest age groups. Changing the assigned value of 0.999 to 0.90 or 1.00 resulted in only small changes in the HALE estimates at birth for both males and females (0.0 to 1.0 years).

**Table 3:** **Results of a sensitivity analysis on the effect of changing HUI3 values on the estimates of health-adjusted life expectancy (HALE) at birth, by diabetes (DM) status and sex, Canada, 2004-2006**^**1**^

**Sex**	**HUI3 weight**	**HALE without DM** **(years)**	**HALE with DM** **(years)**
**Females**	0.999	73.3	62.2
	0.90	72.3	61.3
	1.00	73.3	62.3
**Males**	0.999	70.9	60.1
	0.90	70.9	59.1
	1.00	71.0	60.1

^1^Dataset excluded Quebec, Nunavut, and Northwest Territories.

## Discussion

In this study, LE and HALE were estimated for Canadians with and without diabetes by sex and 19 five-year age intervals using a period life table method. Period life tables provide a convenient approach for summarizing mortality data. Benefits of the life table approach are that HRQOL information is easily integrated and reference to an external standard population is not required. The estimates of LE and HALE were based on the mortality and morbidity experience of Canadians with and without diabetes for the period of 2004 to 2006 (mortality) and 2000 to 2005 (morbidity). Therefore, they should be treated as descriptive cross-sectional statistics based on the past experience of the population rather than as predictive, as the mortality and morbidity experience will change with time.

The results show that LE decreases with increasing age and that the decline is faster for LE than HALE. To explain this observation, the age gradient in the HUI3 scores and mortality rates were compared, revealing that mortality rates increased faster than the HUI3 scores declined with increasing age. The results also confirm that in Canada the LE for females is higher than for males, a direct result of higher mortality among men. This is also true for populations of people with diagnosed diabetes (Table 1).

To evaluate the impact of diabetes on health, the number of years and the proportion of life spent in poor health were assessed and compared between populations of people with and without diabetes (Table 2). The difference in the number of years spent in poor health between populations of people without and with diabetes was not statistically significant for all ages. Table 2 shows the estimated number of unhealthy years with confidence intervals for males and females at selected ages. A test for the difference in the estimated number of years spent in poor health for the diseased and nondiseased populations confirmed statistical significance (p-value <0.05) for females at birth and those who are 70 years and older. This test was also statistically significant for 0- to 19- and 25- to 49-year-old males. Females with diabetes at birth had a significantly greater number of years spent in poor health than their counterparts without diabetes. The reverse pattern was found in women who were 70 years and older; those without diabetes had a significantly greater number of unhealthy years than the corresponding females with diabetes. However, these results, along with the pattern of decline in HUI3 estimates in female populations with and without diabetes 55 years and older, may be misleading due to a data limitation. The CCHS data used in the study do not include persons who lived in institutions. The HRQOL estimates for women in the oldest age groups, as reported by Berthelot et al., could be up to 30% lower if those women who lived in institutions were included [28]. HUI3 could be even lower for persons with diabetes because they might be overrepresented in institutions. In addition, women with diagnosed diabetes receive better health care when they visit physicians and gain advice on how to maintain good health, as opposed to women who have not been diagnosed [1].

Males with diabetes from birth up to 49 years, excluding 20- to24-year-olds, had a significantly greater number of years spent in poor health than their counterparts without diabetes. The reverse pattern (similar to females 70 years and older) was also found for 65- to 79-year-old males, but the difference was not statistically significant. Therefore, it could be concluded that diabetes affects both longevity and quality of life (to some extent). The relationship was different among males and females of different ages and was associated with a significant reduction in the number of healthy years among females at birth and among males younger than 50 years.

A further comparison of female and male populations in the study revealed that females spent more years and a greater portion of life in poor health than did males, confirmed by a large body of evidence [26,27,29]. It was also observed that the number of years spent in poor health decreased and the portion of life a person spent in poor health increased with increasing age. This was true for both sexes, among people with and without diabetes. For example, at birth, females with diabetes spent 17% of their life in poor health, and the percentage gradually increased to 36% by the age of 80. This age gradient in relative differences varied from 14% to 37% for females without diabetes. The same pattern was observed for males, but the proportions were smaller. Females with diabetes lived shorter lives and spent an even greater portion of their lives in poor health than did females without diabetes. The same applies for males.

To evaluate the impact of diabetes on longevity and the number of healthy years, the loss in LE and HALE associated with diabetes was estimated and presented in Table 2. It was observed that females in the age interval of 0 to 54 years had a slightly greater number of years of life lost in HALE than in LE. The reverse pattern was identified for females 55 years and older. The loss in LE was greater than the loss in HALE. For males, the loss in HALE was greater than the loss in LE for all ages excluding 65- to 79-year-old males for whom the reverse pattern was also observed.

In a similar study on hypertension [30], 55-year-old females had a 1.5 year loss in LE and 2.0 year loss in HALE as compared with their counterparts with diabetes who experienced greater losses of 6.0 years and 5.8 years, respectively. Males with hypertension who were 55 years old had a 2.1 year loss in LE and 2.7 year loss in HALE, compared with their counterparts with diabetes who also experienced greater losses of 5.0 years and 5.3 years, respectively. The impact of diabetes on the loss of LE and HALE was greater than the impact of hypertension.

The loss in LE presented in this study is conceptually similar to the life-years lost reported by Narayan et al. [7] for the US population. But because there are differences in the methods of calculation of loss in LE and life-years lost, results may not be directly compared. For example, in our study, the loss in LE estimated for 10-year-old females with diagnosed diabetes was 10.1 years (result not shown), and the life-years lost reported by Narayan et al. was 19.0 years. Notably, the mortality rate ratios [31] used in the Narayan paper were for North Dakota and for a period 10 years earlier than our study. As another example, our loss in HALE estimated for 10-year-old females with diabetes was 10.9 years, and the corresponding QALYs lost in the study by Narayan et al. was 32.8 years. The difference is attributed to a number of differences in the methods of calculation of loss in HALE and QALYs lost. First, the loss in HALE in our study is defined as the difference between HALE for people without diabetes and HALE for people with diabetes. We deduced from the Narayan et al. paper that the QALYs lost are the difference between LE for people without diabetes and QALYs for people with diabetes. Second, in our HALE calculations we applied age-specific HUI3 weights that vary across all age groups. The mean HUI3 for all ages in our data was 0.825 for people with diabetes and 0.872 for people without diabetes. A constant quality of life weight of 0.75 [32] was used for all age groups in the QALYs lost calculations [7].

To quantify the potential gain in LE (or HALE) if hypothetical eradication of diagnosed diabetes was possible, the difference in LE (or HALE) between the population without diabetes and total population was calculated and analyzed. This method, referred to as disease-deleted in this study, is different than the traditional cause-deleted approach [2,3,6]. The principal difference between these two approaches is how mortality rates are adjusted to represent the mortality experience of a diabetes-free population. For the cause-deleted approach the number of deaths categorized as “caused” by diabetes are subtracted from the numerator and the denominator is the total population. This adjustment guarantees that the cause-deleted mortality rates will be lower than the rates for the total population. For the disease-deleted approach all prevalent cases of diabetes (and their deaths) are removed and mortality rates are calculated. Thus, the numerator is the number of deaths among people without diagnosed diabetes and the denominator is the number of people in the population without diagnosed diabetes. It more accurately represents the mortality experience of a population free of diagnosed diabetes. The cause-deleted method works well for diseases with poor survival like myocardial infarction, some types of cancer, or infectious diseases where the underlying cause of death is clear. But it does not work very well for diseases such as diabetes that are associated with comorbidity and manifest themselves in many different causes of death [33]. Using the life expectancy among people without diagnosed diabetes instead of diabetes-deleted life expectancy [2,3,6] (or to be exact diabetes-specific death deleted life expectancy) avoids this problem and allows a more accurate assessment of the diagnosed diabetes burden. Although this method more accurately represents the mortality experience of a population free of diagnosed diabetes, it does not accommodate for competing disease risk factors.

According to this study, the gain in LE at birth after the hypothetical elimination of diagnosed diabetes was 1.4 years for females and 1.3 years for males. The corresponding gain in HALE was 1.2 and 1.3 years for females and males, respectively. This indicates that diabetes is an important disease burden in Canada. These estimates were greater than estimates of the gain in LE and HALE after removing only diabetes-specific death reported by Manuel et al. [5], but the pattern was similar. According to Manuel [2], an expansion of morbidity is evident when years of HALE gained were less than the years gained in life expectancy. In this study, the gain in HALE was less than the gain in LE for males and females of all ages, but the difference between the gain in LE and the gain in HALE was not large, which is consistent with other studies. Although diabetes is an important disease burden, it does not affect morbidity to a great extent. The ratio of HALE to LE reported by Manuel et al. [4,5] for Ontarians with and without diabetes implies the similar conclusion. This ratio quantifies a portion of life people spent in a healthy state. It was estimated to be 0.91 for males without diabetes and 0.90 for males with diabetes. The ratio of HALE to LE was 0.89 for females in both populations. This evidence suggests that impact of diabetes on length of life is similar or slightly smaller than the impact on years of healthy life. LE and HALE for the same population share the same mortality and are highly correlated (r ≈ 1).

Type 1 and type 2 diabetes were not distinguished in this study due to data limitations. However, the majority of cases of diabetes were type 2. There is growing evidence that type 2 diabetes and its complications can be prevented through the reduction of key risk factors such as obesity and physical inactivity. Evidence from the most recently published US study [8] suggests that the lifetime risk of developing diabetes increases over time, thereby decreasing diabetes-free life expectancy. According to the authors, this decrease is closely related to increased obesity prevalence, especially in youth. Type 2 diabetes, previously known as an adult’s disease, is more often seen among children in recent years [34]. The pattern of obesity in Canada is similar to the US and therefore the findings in this research may be applicable to Canada as well. Temporal increases in obesity and diabetes prevalence in Canada may result in decreasing diabetes-free LE and increasing LE with diabetes. Earlier onset of diabetes will likely contribute to decreases in LE and HALE for people with diabetes. However, improvements in diabetes care and further decreases in mortality rates will play the opposite role. The impact of increased obesity and diabetes prevalence and achievements in diabetes care requires further investigation. Therefore, it is important to track changes in both LE and HALE and to monitor the gap between those measures.

The results in this study could be biased toward the null because it was limited to Canadians with or without diagnosed diabetes (those who have been identified by a health professional). Using fasting blood samples collected in the 2007 to 2009 Canadian Health Measures Survey (CHMS), the magnitude of undiagnosed diabetes in Canada was estimated as 0.9% (95% CI: 0.5%-1.4%) of the Canadian population aged 6 years and older (PHAC. Unpublished analysis using 2007–2009 CHMS data. 2011). Therefore, it is possible that those who have been falsely identified by the CCDSS as having diabetes may have had prediabetes.

Another limitation was that the potential gain in HALE might be overestimated due to using the disease-deleted method for this paper. Hypothetically, if diagnosed diabetes could be eliminated, the health status of those people who were living with diagnosed diabetes would likely be lower than of those people who have never had diagnosed diabetes. As a result, the gain in HALE estimated in this study should be somewhat lower than reported.

In addition, the true values of HALE would be somewhat lower than reported because data for residents of long-term care facilities were unavailable. Misclassification of diabetes status was present in both the survey data and the surveillance system data we used. In the CCHS, misclassification can be due to self-reporting bias, generally a tendency to underreport the true disease status. In the CCDSS, misclassification toward the nondiabetic status can be present in geographic areas where data were incomplete. Areas with a larger proportion of salaried physicians provide the least complete data, which results in identifying fewer individuals with disease. Consequently, the disease status concordance between the two data sources varies by province and territory [35,36]. Linkage of these two data sources (CCDSS and CCHS) would provide a method to reduce the self-reporting bias and the misclassification error.

In summary, this paper describes the method used by the Public Health Agency of Canada to calculate life expectancy and health-adjusted life expectancy among Canadian adults with and without diabetes, based on mortality data for the period from 2004 to 2006 and morbidity data for the period from 2000 to 2005. Our work shows that it is possible to calculate HALE for all Canadians and for subpopulations with this particular chronic condition. The results of the study confirm that diabetes is an important disease burden in Canada and it has various impacts for female and male populations of different ages. Our method can be adapted for calculations for other chronic conditions in order to monitor the gap between LE and HALE so that health professionals can assess the impact on good health and revise programs and policies, if warranted.

## Competing interest

The authors declare that they do not have competing interests.

## Authors’ contributions

LL carried out the analysis, contributed to the interpretation of the results, and drafted the initial manuscript. CW contributed to the interpretation and discussion of the results, drafted the article, and revised it. JE and BCKC reviewed and revised the drafts. All authors contributed to the writing of the paper and approved the final version. All authors read and approved the final manuscript.
